# Endoscopic or Follow-up Treatment for Gastric Indeterminate Tumors Is the Preferred Method of Management

**DOI:** 10.3389/fonc.2022.947810

**Published:** 2022-07-13

**Authors:** Jun Xiao, Chao Yu, Jing Chen, Renhu Sun, Hailin Jin, Chunyang Liu, Yaohui Wang, Zhiguang Sun

**Affiliations:** ^1^ Digestive Endoscopy Center, Jiangsu Province Hospital of Chinese Medicine, Affiliated Hospital of Nanjing University of Chinese Medicine, Nanjing, China; ^2^ Department of Pathology, Jiangsu Province Hospital of Chinese Medicine, Affiliated Hospital of Nanjing University of Chinese Medicine, Nanjing, China; ^3^ Second Clinical School of Nanjing University of Chinese Medicine, Nanjing, China

**Keywords:** biopsy, early gastric cancer, gastric indeterminate tumors, endoscopic submucosal dissection, narrow band imaging

## Abstract

**Background:**

Endoscopic forceps biopsy (EFB) lacks precision in diagnosing indeterminate tumors. When the presence of early gastric cancer (EGC) is macroscopically suspected, but biopsy pathology fails to give a diagnosis of neoplasia, it causes problems in clinical management. The purpose of this study was to discuss the outcome of gastric indeterminate tumors and the clinical factors associated with predicting EGC.

**Methods:**

The medical records of 209 patients diagnosed with gastric indeterminate neoplasia by biopsy forceps were retrospectively studied. Initial endoscopic findings were analyzed and predictors of EGC were evaluated.

**Results:**

The final pathological diagnosis in 209 patients included adenocarcinoma (*n* = 7), high-grade intraepithelial neoplasia (*n* = 11), low-grade intraepithelial neoplasia (*n* = 21), and non-neoplastic lesion (*n* = 170). Multivariate analysis showed that older age (OR = 1.78; 95% CI = 1.17–2.71; *p* = 0.008), patients undergoing narrow band imaging (NBI) (OR = 3.40; 95% CI = 1.37–8.43; *p* = 0.008), and surface erosion (OR = 3.53; 95% CI = 1.41–8.84; *p* = 0.007) were associated with the upgraded group, and were significantly associated with risk. Univariate logistic regression analysis showed that among patients with NBI, the presence of demarcation line (DL) (OR = 24.00; 95% CI = 4.99–115.36; *p* < 0.0001), microvascular (MV) pattern irregularity (OR = 9.129; 95% CI = 2.36–35.34; *p* = 0.001), and the presence of white opaque substance (WOS) (OR = 10.77; 95% CI = 1.14–101.72; *p* = 0.038) were significant risk factors.

**Conclusions:**

For gastric indeterminate tumors, older patient age, lesion surface with erosion, clear DL visible under NBI observation, presence of WOS, and irregular MV pattern are suggestive of the high possibility of neoplasia and need to be focused on and may benefit more from endoscopic resection treatment as opposed to simple endoscopic follow-up.

## Introduction

Gastric cancer (GC) causes nearly 103 million new cases and 782,685 deaths worldwide each year (1). In China, GC is one of the most frequent digestive system malignancies. GC deaths in China account for about 50% of global GC deaths, and more than 40% of new cases occur in China (2, 3). The advancement of therapeutic endoscopy, especially the advent of endoscopic submucosal dissection (ESD), has allowed us to treat early gastric cancer (EGC) without lymph node metastases using endoscopic resection (ER) rather than surgical stomach removal in recent years. Endoscopic treatment is seen to be superior to surgical resection because it is less invasive, is less expensive, and provides a higher quality of life (4).

Although GC incidence has decreased dramatically globally over the last half-century, GC has remained a global health problem. The International Agency for Research on Cancer (IARC) of the World Health Organization (WHO) recently released the latest global cancer burden data for 2020, and GC has risen to third place in terms of new cancer cases and deaths in China (5). As a result, in nations with a high incidence of GC, such as China, early identification of EGC is critical. Early gastric cancer screening initiatives at the national level, as well as early gastric cancer screening programs in high-risk locations, have been established in China. The use of screening endoscopy has raised the likelihood of an early diagnosis of superficial GCs and EGC. In recent years, ESD has been clinically superior to surgical gastrectomy. ESD is preferable to surgical gastrectomy in clinically node-negative EGC (4). In the majority of superficial gastric lesions, total resection can be performed effectively (6). Increased screening endoscopy has also increased in endoscopic forceps biopsies, with endoscopic forceps biopsy (7) frequently used to diagnose stomach indeterminate neoplasia (type II lesion of the Vienna classification), although endoscopic forceps biopsy is the most accurate method of confirming gastric tumors and plays a critical role in treatment selection.

However, a forceps biopsy is not always sufficient to make a conclusive diagnosis. While endoscopic lesions appear to be highly malignant, pathology reports for dysplasia (type II lesions) may be ambiguous due to the difficulty in diagnosing atypical epithelial or borderline lesions due to their regenerative or tumorigenic essence (8). If the pathologist is unable to differentiate between neoplastic and non-neoplastic lesions, endoscopic surveillance is indicated for gastric indeterminate tumors, according to the updated Vienna classification (7). If malignant endoscopic findings are suspected, however, total ER may be preferable to endoscopic follow-up.

Although several previous studies have reported histologic discrepancies between endoscopic biopsy and post-treatment pathologic histology of between 2% and 49% (8–11), few studies have examined which endoscopic features contribute to histologic discrepancies in gastric indeterminate tumors. The purpose of this study was to discuss the outcome of indeterminate gastric tumors and the ability to forecast the clinical factors associated with EGC.

## Methods

### Patients

From August 2014 to September 2018, patients visited the Gastrointestinal Endoscopy Center of Jiangsu Province Hospital of Traditional Chinese Medicine for ESD of 983 cases of gastric intraepithelial lesions. During the study period, ESD was used to treat 209 lesions with a preoperative biopsy pathological diagnostic of the gastric indeterminate tumor.

### ESD Procedure

A single-channel endoscope (GIF-H260 or GIF-H260Z; Olympus Optical Co., Ltd., Tokyo, Japan) was used for a diagnostic endoscopy. All ESD procedures were performed on hospitalized patients while sedated with propofol using a conventional one-channel endoscope (GIF-Q260J; Olympus Optical Co., Tokyo, Japan). After identifying the lesion, we injected normal saline containing epinephrine and indigo carmine into the submucosal layer to elevate it above the muscularis propria; we then performed a circular incision and dissection using a needle knife (KD-610L, Olympus Optical Co., Tokyo, Japan). Finally, hemoclips or hemostatic forceps were used to control the bleeding or exposed vessels. To prevent problems such as delayed bleeding or perforation, all patients were instructed to fast for 48 h following ESD and were given proton pump inhibitor infusions intravenously. Meanwhile, all patients were prescribed proton pump inhibitors for 4 to 8 weeks following ESD.

### Endoscopic and Pathologic Evaluation

We evaluated baseline parameters and endoscopic findings. Each endoscopic report was analyzed to ascertain the lesions’ maximum diameter and macroscopic appearance. In each case, endoscopic pictures were analyzed. The Paris classification (12) was used to categorize superficial lesions into three broad categories: elevated, flat, and depressed. Additionally, the lesions’ surface redness, erosion, nodularity, ulceration, and position were assessed. Surface redness was described as red staining of the lesion’s mucosal surface in comparison to the adjacent mucosa. The presence of irregularly elevated or nodular mucosa was termed surface nodularity. The Japanese Classification of Gastric Cancer was used to identify the location of the lesions on the stomach (13). The upper, middle, and lower regions of the stomach area are separated into three equal sections in this approach.

Two pathologists assessed all endoscopic forceps biopsy samples and resected tissue slides blinded. To obtain agreement, discordant cases were reevaluated using the multi-headed microscope. Stretching, pinning, and formalin fixation were used to fix the resected specimens. At 2-mm intervals, the fixed specimen was sectioned. According to the Vienna classification, all lesions were categorized as gastrointestinal epithelial neoplasia (7).

### Statistical Analyses

Statistical analyses were performed using SAS software, version 9.2 (SAS Institute, Cary, NC, USA). Data were presented as mean ± SD for continuous variables, or as a percentage for categorical variables. Means and proportions were compared by the Student’s *t*-test and Fisher’s exact test, respectively. We compared concordant and upgraded lesions concerning their clinical and endoscopic characteristics in all patients and subjects who underwent narrow band imaging (NBI) detection, respectively. We also performed univariate and multivariate logistic regression analyses to identify significant endoscopic predictors of histologic upgraded lesions after ESD. A two-tailed *p* < 0.05 was considered statistically significant.

## Results

### Characteristics of the Study Patients

The 209 patients [103 (49.3%) men] had a mean age of 53.8 ± 12.2 years and included 47 (22.5%) patients who underwent NBI examination. The mean lesion diameter and width were 2.02 ± 1.07 cm and 1.71 ± 0.80 cm, respectively. Overall, 165 (79.0%) cases had a reddish lesion color, 91 (43.5%) cases were located in the lower third of the stomach, and 61 (29.2%) cases were on the posterior gastric wall. We classified macroscopic morphology into two types: elevated [113 (54.1%) specimens] and flat [96 (45.9%) specimens]. The prevalence of surface ulceration, nodularity, and erosion was 2.4%, 20.1%, and 34.0%, respectively.

As defined above, we classified two groups according to changes between pre- and post-ESD pathology: concordant and upgraded. We identified 170 (81.3%) in the concordant group (non-neoplastic) and 39 (18.7%) in the upgraded group. Of 39 histopathology-confirmed upgraded cases after post-ESD, low-grade intraepithelial neoplasia, high-grade intraepithelial neoplasia, and adenocarcinoma were observed in 21 (53.8%), 11 (28.2%), and 7 (18.0%) patients, respectively. Of note, 50.2% of the high-grade dysplasia (upgraded) group was confirmed to have adenocarcinoma in the final ESD pathology. The upgraded group, compared with the concordant group, had similar characteristics (*p* ≥ 0.06) except for age, application of diagnostic modality, lesion width, location (short axis) distribution, and the rates for erosion (*p* ≤ 0.02, **Table 1**). Upgraded group patients were older (+7.3 years), had a larger lesion width (+0.34 cm), had higher proportions of NBI examination (46.1% vs. 17.1%), had lesions located in the middle third of the stomach (30.8% vs. 11.8%), and had higher rates for erosion (51.3% vs. 30.0%) (*p* ≤ 0.02, **Table 1**).

**Table 1:** Characteristics of the study patients between concordant and upgraded groups.

Characteristics	All (*n* = 209)	Concordant (*n* = 170)	Upgraded (*n* = 39)	*p*
Age, years	53.8 ± 12.2	52.5 ± 12.8	59.8 ± 7.0	**<0.001**
Gender, *n* (%)				0.78
Male	103 (49.3)	83 (48.8)	20 (51.3)	
Female	106 (50.7)	87 (51.2)	19 (48.7)	
Diagnostic modality, *n* (%)				**<0.0001**
WLI	162 (77.5)	141 (82.9)	21 (53.9)	
NBI	47 (22.5)	29 (17.1)	18 (46.1)	
Lesion diameter, cm	2.02 ± 1.07	1.95 ± 1.04	2.32 ± 1.19	0.06
Lesion width, cm	1.71 ± 0.80	1.64 ± 0.77	1.98 ± 0.89	**0.02**
Lesion color, *n* (%)				0.16
Similar color as surroundings	38 (18.2)	35 (20.6)	3 (7.7)	
Reddish	165 (79.0)	130 (76.5)	35 (89.7)	
Whitish	6 (2.8)	5 (2.9)	1 (2.6)	
Location: long axis, *n* (%)				**0.009**
Upper third	86 (41.2)	71 (41.8)	15 (38.4)	
Middle third	32 (15.3)	20 (11.8)	12 (30.8)	
Lower third	91 (43.5)	79 (46.4)	12 (30.8)	
Location: short axis, *n* (%)				0.76
Large curvature	48 (23.0)	37 (21.8)	11 (28.2)	
Lesser curvature	59 (28.2)	49 (28.8)	10 (25.6)	
Anterior gastric wall	41 (19.6)	35 (20.6)	6 (15.4)	
Posterior gastric wall	61 (29.2)	49 (28.8)	12 (30.8)	
Macroscopic morphology, *n* (%)				0.07
Elevated	113 (54.1)	97 (57.1)	16 (41.0)	
Flat/Depressed	96 (45.9)	73 (42.9)	23 (59.0)	
Ulceration, *n* (%)	5 (2.4)	3 (1.8)	2 (5.1)	0.22
Nodularity, *n* (%)	42 (20.1)	31 (18.2)	11 (28.2)	0.16
Erosion, *n* (%)	71 (34.0)	51 (30.0)	20 (51.3)	**0.01**

Data are mean ± standard deviation or number with the percentage in parenthesis. These bold values are all p<0.05, representing statistical significance.

### Analysis of Risk Factors for the Upgraded Group

In univariate logistic regression analyses, older age, patients undergoing NBI examination, larger lesion width, lesion located in the middle third of the stomach, and surface erosion were identified as significant risk factors associated with the upgraded group (OR = 1.78 to 4.17, *p* ≤ 0.02, **Table 2**).

**Table 2:** Multi-variable logistic regression analysis of risk factors for the upgraded group.

Variables	Crude Model		Adjusted Model	
	OR (95% CI)	*p*	OR (95% CI)	*p*
**Age** (+10 years**)**	1.797 (1.265–2.554)	**0.001**	1.779 (1.167–2.712)	**0.008**
**Gender** (women vs. men**)**	0.906 (0.452–1.818)	0.78		
**Diagnostic modality** (NBI vs. WLI)	4.168 (1.977–8.785)	**0.0002**	3.402 (1.374–8.425)	**0.008**
**Lesion diameter** (+1 cm)	1.317 (0.987–1.759)	0.06		
**Lesion width** (+1 cm)	1.589 (1.067–2.365)	**0.02**		
**Lesion color**				
Similar color as surroundings	Reference			
Reddish	3.140 (0.912–10.814)	0.07		
Whitish	2.333 (0.201–27.014)	0.50		
**Location: long axis**				
Upper third	Reference			
Middle third	2.840 (1.147–7.033)	**0.02**		
Lower third	0.719 (0.315–1.639)	0.43		
**Location: short axis**				
Large curvature	Reference			
Lesser curvature	0.686 (0.264–1.787)	0.44		
Anterior gastric wall	0.577 (0.193–1.727)	0.33		
Posterior gastric wall	0.824 (0.327–2.073)	0.68		
**Macroscopic morphology** (Flat/Depressed vs. Elevated)	1.910 (0.942–3.872)	0.07		
**Ulceration** (yes vs. no)	3.009 (0.485–18.651)	0.24		
**Nodularity** (yes vs. no)	1.762 (0.793–3.915)	0.16		
**Erosion** (yes vs. no)	2.456 (1.209–4.988)	**0.01**	3.534 (1.413–8.836)	**0.007**

Values are odds ratio (OR) with 95% confidence interval (CI). The adjusted model was adjusted for age, sex diagnostic modality, lesion location (long/short axis), diameter and width, color, macroscopic morphology, ulceration, nodularity, and erosion. These bold values are all p<0.05, representing statistical significance.

In multivariate analyses, after adjustment for age, sex, diagnostic modality, lesion location (long/short axis), diameter and width, color, macroscopic morphology, ulceration, nodularity, and erosion, older age (OR = 1.78; 95% CI = 1.17−2.71; *p* = 0.008), patients undergoing NBI examination (OR = 3.40; 95% CI = 1.37−8.43; *p* = 0.008), and surface erosion (OR = 3.53; 95% CI = 1.41−8.84; *p* = 0.007) were still significantly associated with the risk of the upgraded group (**Table 2**).

### Subgroup Analysis

**Figure 1:**
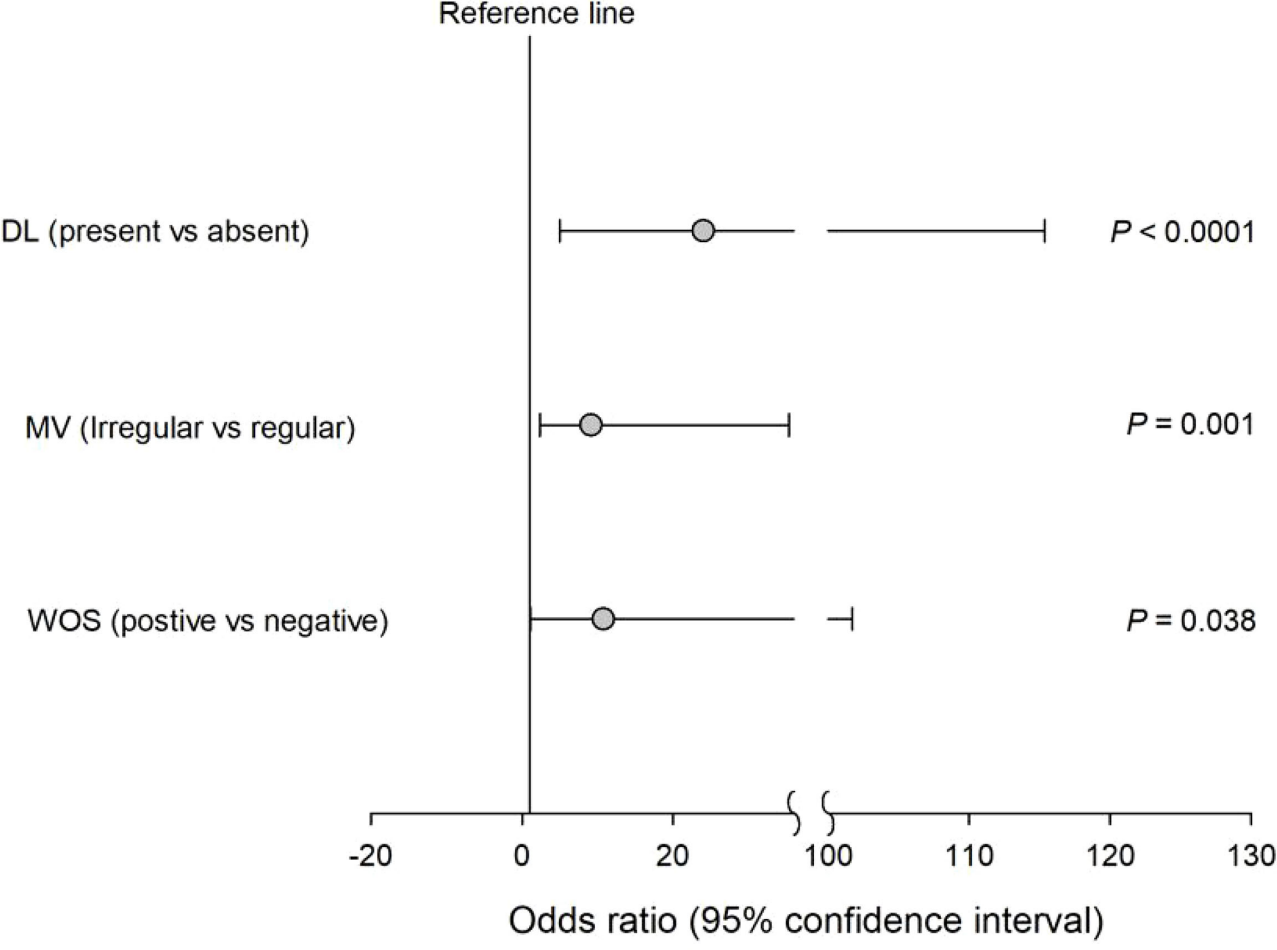
Univariate logistic regression analyses revealed that the presence of DL irregularity of MV and the presence of WOS were significantly associated with the risk of the upgraded group in patients with NBI use.

We finally performed a subgroup analysis to investigate the NBI findings associated with the upgraded group in patients who underwent NBI examination. **Table 3** summarizes the baseline characteristics in these patients between concordant and upgraded groups. The upgraded group, compared with the concordant group, had higher rates for the presence of demarcation line (DL) (83.3% vs. 17.2%), irregularity of MV (72.2% vs. 10.3%), and presence of the white opaque substance (WOS) (27.8 vs. 3.4%) (*p* ≤ 0.02, **Table 3**). Also, univariate logistic regression analyses revealed that the presence of DL (OR = 24.00; 95% CI = 4.99−115.36; *p* < 0.0001), the irregularity of MV (OR = 9.129; 95% CI = 2.36−35.34; *p* = 0.001), and the presence of WOS (OR = 10.77; 95% CI = 1.14−101.72; *p* = 0.038) were significantly associated with the risk of the upgraded group in patients with NBI use (**Figure 1**).

**Table 3:** Characteristics of the study patients using NBI between concordant and upgraded groups.

Characteristics	All (*n* = 47)	Concordant (*n* = 29)	Upgraded (*n* = 18)	*p*
Demarcation line, *n* (%)				**<0.0001**
Absent	27 (49.3)	24 (82.8)	3 (16.7)	
Present	20 (50.7)	5 (17.2)	15 (83.3)	
Microvascular pattern, *n* (%)				**<0.0001**
Regular	30 (63.9)	25 (86.2)	5 (27.8)	
Irregular	16 (34.0)	3 (10.3)	13 (72.2)	
Absent	1 (2.1)	1 (3.5)	0 (0)	
Microsurface pattern, *n* (%)				0.12
Regular	16 (34.0)	13 (44.8)	3 (16.7)	
Irregular	24 (51.1)	13 (44.8)	11 (61.1)	
Absent	7 (14.9)	3 (10.4)	4 (22.2)	
WGA, *n* (%)				0.25
Negative	45 (95.7)	27 (93.1)	18 (100)	
Positive	2 (4.3)	2 (6.9)	0 (0)	
WOS, *n* (%)				**0.02**
Negative	41 (87.2)	28 (96.6)	13 (72.2)	
Positive	6 (12.8)	1 (3.4)	5 (27.8)	

Data are numbers with the percentage in parenthesis. These bold values are all p<0.05, representing statistical significance.

## Discussion

ER methods include ESD and endoscopic mucosal resection (EMR). The overall resection rate and histological complete resection rate of ER were lower than those of gastrectomy, and the recurrence rate and metachronous cancer rate were significantly higher than those of the gastrectomy group (14–18). However, compared with gastrectomy, ER has a shorter hospital stay and a lower complication rate in the treatment of EGC (19–21). However, overall survival was similar for both (22). In conclusion, ER is as effective as gastrectomy while being safer and less costly in EGC patients. ESD is a new treatment method based on EMR, which can resect lesions > 2 cm. Compared with EMR, ESD can accurately resect submucosa lesions under the guidance of endoscopy, and it meets the standard of complete resection (23).

Endoscopic surveillance is recommended in the case of indeterminate gastric neoplasia (type II lesion) or stomach atypical cells (7). Although an endoscopic forceps biopsy is the best approach for diagnosing dysplastic lesions, it can be difficult to rule out definite neoplasia due to the heterogeneous histology of the lesion. In the current study, 18.7% of lesions (39/209) constitute confirmed neoplasia (7 adenocarcinomas, 21 low-grade intraepithelial neoplasia, and 11 high-grade intraepithelial neoplasia). The reasons behind the difficulties in making a correct diagnosis based on the initial biopsy material are debatable. The following are some possible explanations: (1) structural heterogeneity of atypical hyperplasia is difficult to detect in small biopsy specimens; (2) heterogeneity of cancer distribution may result in sampling errors; (3) the limited amount of tissue obtained from small biopsy specimens, combined with the fact that some specific types of cancer originate from the glandular neck, makes effective tissue for biopsy difficult to obtain; and (4) tissue regeneration demonstrating histological changes caused by heterogeneity caused by gastritis (24). As a result, a higher-grade lesion, such as high-grade dysplasia or focally present cancer, may be discovered from the lesion that was previously classified as indeterminate gastric neoplasia *via* endoscopic forceps biopsy. Previous research has demonstrated a histologic discrepancy between forceps biopsy and resected specimens in gastric superficial neoplasia, resulting in an underdiagnosis rate of up to 33.9%–49% (8–10, 25). There are three types of discrepancy: downgraded, concordant, and upgraded. The upgraded discrepancy is the most problematic of the three types. The current study found an 18.7% (39/209) rate of underdiagnosis and pathological progression following ESD, implying that preoperative endoscopic biopsy diagnosis was insufficient to determine the pathological nature of stomach ambiguous tumors.

Numerous earlier investigations have produced comparable findings. The risk factors commonly associated with upgrade discrepancy include lesion size, depressed morphology, ulceration, and whitish discoloration (25–27). Prior research indicated that risk factors for malignant dysplastic alterations included lesion diameters more than 1 cm, depressed appearance, erythematous mucosal change, and surface erosion (26, 28–30). In our research, we found similar outcomes. Older age (OR = 1.78; 95% CI = 1.17−2.71; *p* = 0.008) and surface erosion (OR = 3.53; 95% CI = 1.41−8.84; *p* = 0.007) were significantly associated with risk factors for our upgraded group. Choong-Kyun Noh et al. reported discrepancies between endoscopic forceps biopsy and ESD specimens including upgraded, concordant, and downgraded diagnoses in gastric neoplasms (25). Among these, they found that surface ulceration and depressed lesions were associated with significant risk factors for upgrading. However, due to NBI deficiency in some endoscopic results, their study could not analyze it to answer the function of NBI. This was supplemented somewhat in our investigation. Patients who had NBI examinations were shown to be affiliated with the risk of being upgraded in the current study. In patients with NBI use, the presence of DL (OR = 24.00; 95% CI = 4.99–115.36; *p* = 0.0001), the irregularity of MV (OR = 9.129; 95% CI = 2.36–35.34; *p* = 0.001), and the presence of WOS (OR = 10.77; 95% CI = 1.14–101.72; *p* = 0.038) were all significantly associated with the risk of the upgraded group.

It is critical to choose patients who require ESD to confirm a clear diagnosis in clinical practice, Although endoscopic forceps biopsy outcomes are uncertain, understanding of the endoscopic features predictive of EGC is significant. EGC was found to be substantially associated with surface erosion in the current investigation. Without a doubt, the first endoscopic target biopsy is a critical diagnostic step. As a result, endoscopists are aware of the risk factors for definite neoplasia and conduct extensive examinations and biopsies with care. Endoscopists have made reliable diagnoses of lesions using a variety of approaches. Numerous assistive techniques, such as image-enhanced endoscopy, can supplement the endoscopist’s vision. Recently, image-enhanced endoscopies, such as magnifying NBI endoscopy, were reported to be capable of predicting histologic characteristics of EGC (31) and the histologic severity of gastritis (32). In this study, we discovered that the NBI technique has a greater advantage in predicting gastric indeterminate tumors, particularly when the suspicious lesions have clear borders, irregular microvessels, or WOS, which often indicate a pathological nature of neoplastic lesions, and that the choice of ESD treatment benefits patients more than endoscopic follow-up alone.

There is still a lack of data on the clinical importance of indefinite gastric neoplasia (type II lesion). Accurate dysplasia diagnosis and grading are critical because reported rates of dysplasia progression to GC range from 0% to 73% per year (7, 33). More aggressive management, such as ESD or endoscopic mucosal excision, should be considered instead of follow-up endoscopic biopsy for highly suspicious definite neoplastic lesions for the accurate identification of gastric indeterminate neoplasia by endoscopic forceps biopsy. Because it allows for en bloc resection, ESD is a useful endoscopic method for the treatment of stomach superficial neoplasia. Although the differences in rates of effective en bloc resection, complications, and operation times with ESD between studies are assumed to be due to the devices employed and the operator’s experience, ESD enables a higher en bloc and pathologically complete resection rate and lower local recurrence compared to EMR (34–36). This investigation revealed no significant procedure-related concerns.

Several limitations apply to the current investigation. For starters, this retrospective analysis of stomach ambiguous tumors could have been influenced by selection bias. Second, while we initially saw benefits from using NBI, an image-enhanced endoscope, the operation is closely related to the operator’s experience, and this study was performed on gastric indeterminate tumors, where the heterogeneity of the lesion itself is relatively weak and the features under NBI observation may be less significant. There could be some bias here.

In conclusion, for some individuals, a straightforward follow-up strategy for gastric indeterminate tumor (category 2) lesions is insufficient. Because endoscopic forceps biopsy tissue may not be typical of the full dysplastic lesion, certain differences may exist between endoscopic forceps biopsy samples and resected specimens. According to the findings of this study, higher-grade lesions are more likely to go undiagnosed. In high-risk patients, a simple endoscopic follow-up strategy may miss the opportunity to treat EGC endoscopically. Furthermore, repeated endoscopy with biopsy may be physically, emotionally, and financially stressful for individuals. Precautions should be made in the therapy of patients with ambiguous gastric tumors, especially if the patient is elderly and the lesion surface is erodible. The NBI examination is useful in determining the severity of the lesion. Endoscopic total resection, rather than a simple endoscopic follow-up plan, should be considered for lesions with well-defined margins, irregular microvessels, and the presence of WOS. For lesions with no risk factors, a follow-up endoscopy may be advised. In the future, image-enhanced endoscopy may increase the diagnostic accuracy of stomach indeterminate tumors.

## Limitation

Due to the small sample size, the data may be biased; therefore, more central, prospective, large-sample studies and longer-term follow-ups are needed to validate the findings.

## Data Availability Statement

The original contributions presented in the study are included in the article/supplementary material. Further inquiries can be directed to the corresponding author.

## Ethics Statement

The studies involving human participants were reviewed and approved by the Affiliated Hospital of the Nanjing University of Chinese Medicine. The participants provided their written informed consent to participate in this study.

## Author Contributions

The conception and design of this article were mainly done by JX and ZS. The provision of study materials or patient information was collected and organized by CY, JC, RS, and HJ. Histopathological testing work was done by CL and YW. JX was responsible for manuscript writing. All authors approved the final version of the manuscript.

## Funding

This work was supported by the Jiangsu Province Traditional Chinese Medicine Science and Technology Development Project (2020zx07).

## Conflict of Interest

The authors declare that the research was conducted in the absence of any commercial or financial relationships that could be construed as a potential conflict of interest.

## Publisher’s Note

All claims expressed in this article are solely those of the authors and do not necessarily represent those of their affiliated organizations, or those of the publisher, the editors and the reviewers. Any product that may be evaluated in this article, or claim that may be made by its manufacturer, is not guaranteed or endorsed by the publisher.
